# Transcriptomic Differences Related to Neck Pain in Patients with Oropharyngeal Squamous Cell Carcinoma

**DOI:** 10.3390/genes16111277

**Published:** 2025-10-28

**Authors:** Monica A. Wagner, Charles Djordjevic, Marci L. Nilsen

**Affiliations:** 1 Frances Payne Bolton School of Nursing, Case Western Reserve University, Cleveland, OH 44106, USA; 2 Department of Otolaryngology, University of Pittsburgh, Pittsburgh, PA 15260, USA; mlf981@pitt.edu

**Keywords:** pain, transcriptomics, patient-reported outcomes, oropharyngeal squamous cell carcinoma, head and neck cancer

## Abstract

**Background/Objectives:** Neck-specific pain and disability are common and burdensome for survivors of oropharyngeal squamous cell carcinoma (OPSCC), yet the biological mechanisms underlying these symptoms remain poorly understood. While patient-reported outcomes (PROs) offer valuable insight into pain and function, their limited integration with molecular data restricts the development of targeted interventions. The purpose of this study was to explore transcriptomic differences associated with neck pain and disability in OPSCC survivors. **Methods**: Bulk RNA sequencing was performed on blood samples collected from OPSCC survivors either pre-radiation or more than one year post treatment. DESeq2 was used to determine differentially expressed genes between survivors reporting no versus any neck-related pain, as measured by the validated Neck Disability Index. Ingenuity Pathway Analysis was used to explore interaction among the genes. **Results**: We identified 24 significantly differentially expressed genes (adjusted *p* < 0.05) linked to hematopoietic, immune, and neuronal functions. Pathway analysis of the top 50 differentially expressed genes revealed overlap in interferon signaling, iron homeostasis, and blood cell development, suggesting molecular connectivity in hematologic and immunologic disease, cellular movement, and connective tissue disorders. **Conclusions**: These findings suggest the existence of molecular phenotypes associated with patient-reported neck pain and disability in OPSCC survivors and highlight the importance of integrating PROs with molecular profiling to better understand survivorship burden.

## 1. Introduction

Head and neck cancer (HNC) represents a growing public health concern, accounting for 3–4% of all cancers in the United States (US) and affecting nearly 70,000 individuals annually [1,2]. Despite advancements in treatment and prevention, the overall incidence of HNC continues to rise, with a predicted 30% annual increase by 2030 [3,4]. Oropharyngeal squamous cell carcinoma (OPSCC) is a distinct and increasingly prevalent HNC subtype, primarily driven by human papillomavirus (HPV) infection [5]. HPV-positive OPSCC now accounts for over 70% of all OPSCC cases and is expected to represent more than half of all HNC diagnoses in the US by 2030 [6]. As the number of HNC survivors increases, evaluating quality of life (QOL) and functional outcomes has become a critical component of survivorship care.

Neck pain and disability are highly prevalent among HNC survivors and can persist for years after treatment [7,8,9]. Studies show that approximately 55% of HNC survivors experience some degree of neck disability [7]. Additionally, persistent pain, including that in the neck area, is reported in nearly 31% of survivors, even months or years after completing treatment [10]. This pain and disability are often linked to treatment-related factors such as surgery, radiation, and neck dissection and can significantly impact long-term QOL [11].

Patient-reported outcomes (PROs) offer direct, patient-centered insights into symptoms such as pain, fatigue, and anxiety, and their impact on daily functioning and QOL, factors that cannot be measured through clinical observation [12,13]. Subjective PRO measures correlate with objective indicators of dysfunction and, due to their low cost and ease of implementation, are valuable tools in clinical practice [8]. PROs facilitate early detection and intervention, support personalized care, and contribute to more informed clinical decision-making, and more efficient resource allocation [14]. Assessing the functional limitations associated with neck pain and disability in the OPSCC community is essential for clinical decision-making, treatment planning, and outcome evaluation [15].

Despite growing recognition of the importance of survivorship outcomes in OPSCC, significant gaps remain in our understanding of the biological and clinical underpinning of neck pain and disability in this population. The molecular mechanisms driving these symptoms are poorly characterized, limiting the development of targeted interventions [16,17,18]. Current research rarely integrates clinical symptomatology, PROs, and molecular data, an approach that could yield more comprehensive insights into disease burden and survivorship trajectories. Additionally, pain features specific to the head and neck region are often under-characterized, despite their relevance to functional impairment and QOL in cancer survivors [19,20]. Addressing these gaps has the potential to transform clinical care by enabling earlier identification of high-risk patients, guiding personalized symptom management strategies, and improving long-term functional outcomes [21,22].

Understanding the biological basis of symptom burden in OPSCC is critical to improving survivorship outcomes. While PROs offer valuable insights into pain, fatigue, and function impairment, they are rarely integrated with molecular data, limiting their utility in guiding targeted interventions. Transcriptomic profiling provides a dynamic snapshot of gene expression that reflects real-time cellular responses to disease and treatment, offering a powerful tool to explore the mechanisms underlying symptom biology. Linking PROs to transcriptomic data enables the identification of molecular pain signatures and symptoms associated pathways, which may serve as novel therapeutic targets. This integrative approach has the potential to advance personalized care by stratifying patients based on biological risk, tailoring symptom management strategies, and ultimately improving long-term functional outcomes.

The purpose of this study was to investigate transcriptomic differences associated with patient-reported neck pain and disability in OPSCC survivors via self-reported responses to the Neck Disability Index (NDI). By integrating gene expression data with patient-reported symptom profiles, we sought to characterize molecular mechanisms underlying symptom development and identify potential therapeutic targets. This work lays the foundation for biologically informed, personalized interventions that improve symptom management and long-term QOL for OPSCC survivors.

## 2. Materials and Methods

### 2.1. Sample and Setting

The current study capitalizes on clinical, sociodemographic, and gene expression (transcriptome) data collected from participants seen at the UPMC Head & Neck Cancer Survivorship Clinic. The sample consisted of 51 participants diagnosed with OPSCC, an anatomical subsite of head and neck cancer. Participants were able to speak and read English, had completed at least 8 years of education, and were scheduled to or had already received radiation. Participants were characterized according to clinician-assigned grades of radiation-induced fibrosis using the National Cancer Institute Common Terminology Criteria for Adverse Events (CTCAE). Those assessed prior to radiation were classified as Grade 0 (no fibrosis). Participants with Grade 1 fibrosis (asymptomatic or mild symptoms; no intervention indicated) were grouped as ‘mild’, while those scored with Grades 2 (moderate symptoms; minimal, local or noninvasive intervention indicated) or 3 (severe or medically significant symptoms, intense intervention indicated) were grouped as ‘moderate/severe’. All stages of cancer were included, based on the American Joint Commission on Cancer (AJCC) TNM classification system [23]. Informed consent was obtained from all participants. This study was approved by the University of Pittsburgh Institutional Review Board.

### 2.2. Sociodemographic and Clinical Characteristics

Sociodemographic and clinical characteristics were obtained via medical record review. Sociodemographic factors included age (years), sex (female, male), race (White, Other), tobacco use (yes, no), and alcohol intake (yes, no). Clinical data included tumor stage (T1/T2, T3/T4), radiation dose (Gray), time since last radiation (months), whether they received adjuvant chemotherapy, and National Area Deprivation Index (ADI). ADI is a measure of neighborhood disadvantage that is determined by entering the complete home address into the Neighborhood Atlas database [24,25]. National ADI ranges from 1 to 100, with higher values indicating greater neighborhood disadvantage.

### 2.3. Evaluation of Neck Pain

Neck pain and disability were assessed via the NDI [26]. The NDI is the most commonly used self-report tool for neck pain and has been shown to have both internal consistency and a high degree of test/retest reliability [27,28,29]. NDI scores are determined based on degree of disability (none: 0 to 4, mild to moderate: 5 to 24, and severe to complete: 25 to 50) [30]. The 10-question NDI assesses: pain intensity, personal care, lifting, reading, headaches, concentration, work, driving, sleeping, and recreation. Items are scored on a scale from 0 (no disability) to 5 (complete disability). For this study, NDI was dichotomized into two groups: none (0 to 4) or any (5 to 50) disability. This dichotomization has been used in previous studies [13,31]. We analyzed gene expression differences between individuals with any versus no neck disability to identify associated genes.

Participants also self-reported how much general pain they were experiencing from 0 (no pain) to 10 (worst pain imagined) at time of assessment. NCCN guidelines [32] classify pain into the following categories: no pain (0), mild pain (1 to 3), moderate/severe pain (4 to 10). Pain was also dichotomized into no pain (0) or any pain (1 to 10) for this study. We controlled for the presence or absence of pain in our gene expression analysis.

### 2.4. Peripheral Blood Collection and RNA Extraction

All samples were collected using standardized protocols. RNA was extracted from intracellular peripheral blood cells preserved in PAXgene Blood RNA Tubes (PreAnaltiX, Qiagen/BD, Hombrechtikon, Switzerland) and stored at −70 °C until processing. RNA was extracted using the PAXgene Blood RNA Kit (Qiagen), following the manufacturer’s protocol. RNA concentration and purity were assessed using the Agilent 2100 Bioanalyzer (Agilent Technologies, Santa Clara, CA, USA). Samples with an RNA integrity number (RIN) ≥ 7.0 were selected for sequencing. Batch effects were controlled during sample preprocessing.

### 2.5. Library Preparation and Sequencing

RNA libraries were prepared by the University of Pittsburgh Genomics Core using the Illumina (Santa Clara, CA, USA) TruSeq Stranded mRNA Library Prep Kit, which includes poly-A selection. The poly-A selections step further enriched for mature mRNA transcripts from cellular sources. Free circulating RNA (e.g., cell-free RNA or exosomal RNA) was not targeted or captured in this workflow. Libraries were quantified using Qubit fluorometry (Thermo Fisher Scientific, Waltham, MA, USA) and pooled for sequencing on an Illumina NovaSeq 6000 Platform, generating paired-end 150 bp reads with a target depth of ~30–40 million reads per sample.

### 2.6. Bioinformatic Processing and Quality Control

Raw sequencing data were processed using CLC Genomics Workbench v24 (Qiagen, Santa Clarita, CA, USA) for initial quality control, trimming, and alignment to the GRCh38/hg38 human reference genome. Gene level count data were exported and analyzed in R (v4.1.2) using DESeq2 (v1.34.0, Bioconductor, Boston, MA, USA). The design formula used for DESeq2 was ‘~Fibrosis Level + Pain + Neck Disability’. In this analysis, DEseq2 controlled for Fibrosis Level and Pain while testing the effect of Neck Disability. We included relevant covariates in the design formula to account for potential batch effects. Genes with fewer than 10 total counts across all samples were filtered prior to normalization to reduce noise and improve statistical power. Wald test *p*-values were adjusted using the Benjamini–Hochberg method to control the false discovery rate (FDR). Genes with an adjusted *p*-value (FDR) ≤ 0.05 were considered statistically significant. A heatmap of the top 50 differentially expressed genes using the unadjusted *p*-values (*p*-value ≤ 0.05) was also generated to explore relative differences and help identify trends in differential expression that many warrant further investigation.

### 2.7. Pathway Enrichment Analysis

To identify biological processes associated with neck pain and disability in OPSCC, we performed pathway enrichment analysis using Ingenuity Pathway Analysis (IPA, Qiagen, Germantown, MD, USA) on the set of differentially expressed genes. Differentially expressed genes identified using a *p*-value threshold of ≤0.10 and a normalized fold change of ±1.5 were used for pathway analysis in IPA. This filtered gene set also served as the background reference gene set for statistical enrichment. Pathway enrichment significance was determined using the right-tailed Fisher’s exact test to determine overrepresentation (i.e., pathways with more molecules from the reference set than expected). The Benjamini–Hochberg (B-H) FDR correction was applied to control for false positives [33].

### 2.8. Statistical Analysis

Statistical comparisons across pre-radiation, mild and moderate groups were conducted using IBM SPSS Statistics Version 29 (IBM Corp, Armonk, NY, USA). For the descriptive analysis, mean and standard deviation were calculated for continuous variables; frequency counts and percentage were calculated for categorical variables. The Pearson correlation coefficient (r) was calculated between the NDI values and patient-reported pain scores. A negligible pain score was defined as an absolute value of r between 0 and 0.29, a low correlation between 0.3 and 0.49, a moderate correlation between 0.5 and 0.69, and a strong correlation between 0.7 and 1.0. We used basic statistical tests to compare fibrosis cohorts across the variables in Table 1. Continuous variables were assessed for normality using the Shapiro–Wilk test. Depending on distribution, either one-way ANOVA (for normally distributed data) or Kruskal–Wallis test (for non-parametric data) were applied. Categorical variables were analyzed using Chi-square tests or Fisher’s exact tests when appropriate. A *p*-value threshold of <0.05 was used to determine statistical significance.

## 3. Results

### 3.1. Participant Characteristics

Sociodemographic, clinical, and transcriptome data were available for 51 participants divided into clinical cohorts of no fibrosis (n = 13, 25.5%), mild fibrosis (n = 20, 39.2%), and moderate fibrosis (n = 18, 35.3%; Table 1). Patient characteristics reveal that cohorts had no statistical differences in age, race, sex, tobacco use, alcohol use, tumor stage, or national ADI. There were statistical differences in marital status (*p* = 0.034), neck disability (*p* = 0.003), and self-reported pain (*p* < 0.001).

There was no statistical difference between the mild and moderate/severe fibrosis cohorts in radiation dose or elapsed time (in months) since last radiation treatment. There was also no difference between the two cohorts in number of participants who also received chemotherapy. There was a strong correlation (*r* = 0.779) between reported pain score and NDI scores.

### 3.2. Differential Gene Expression

**Figure 1:**
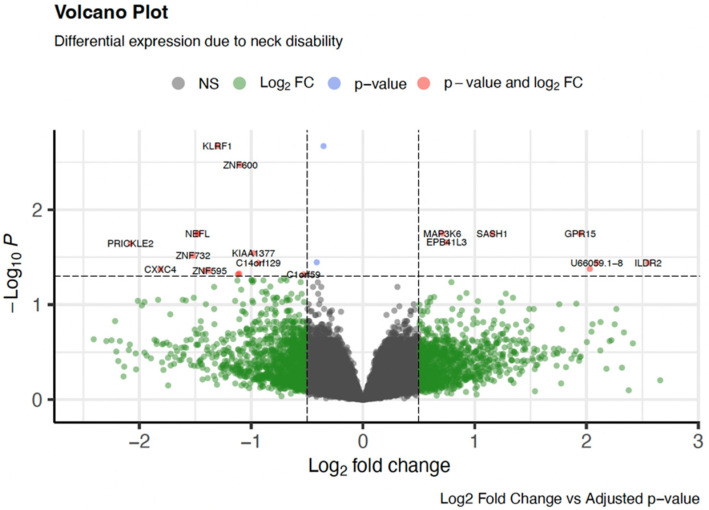
A volcano plot illustrating differential gene expression associated with neck disability. Each point represents a gene, plotted by it log2 fold change (x-axis) and −log10 adjusted *p*-value (y-axis). Genes with both statistically significant expression changes and substantial fold changes are highlighted in red, while those with either significant *p*-values (blue) or fold changes (green) alone are also marked. Non-significant genes are shown in grey. Notable genes such as *KLRF1*, *ZNF600*, *PRICKLE2*, and *NEFL* are labeled for emphasis. This visualization enables rapid identification of genes most affected by neck disability, supporting downstream pathway and functional enrichment analysis.

Differential expression analysis comparing patients with no neck disability (NDI = none; n = 39) and those with any level of disability (NDI = mild/moderate/severe; n = 12) identified 24 significantly differentially expressed genes (adjusted *p* < 0.05; Table 2). A volcano plot (Figure 1) illustrates the distribution of gene expression changes highlighting genes with both statistical significance (adjusted *p* < 0.05) and fold change (|log_2_FC| > 0.5).

**Figure 2:**
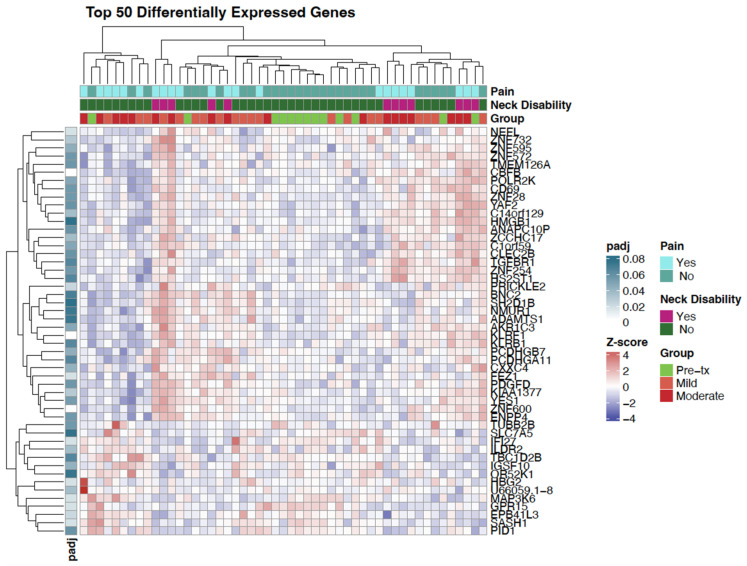
Heatmap showing the expression profiles of the top 50 DEGs (*p* ≤ 0.05) across all samples. Rows represent individual genes, and columns represent samples grouped by clinical annotations: Fibrosis Level (No Fibrosis, Mild Fibrosis, Moderate/Severe Fibrosis), Pain Category (None, Mild/Moderate-Severe), and NDI category (None, Mild/Moderate-Severe). Gene expression values were z-score-normalized, with red indicating higher expression and blue indicating lower expression. Hierarchical clustering was applied to both genes and samples using Euclidean distance and complete linkage. The adjusted *p*-value is represented on the left for visualization but the genes in this heatmap were chosen based on *p*-value. This visualization highlights differentiated expression patterns corresponding to clinical phenotypes in HNC patients and provides a broader visual overview of transcriptomic variation across the sample.

To further explore expression patterns, a heatmap of the top 50 differentially expressed genes was generated (Figure 2). This visualization revealed notable clustering of samples related to neck disability, with several genes showing consistent up- or down-regulation in participants reporting any neck disability.

### 3.3. Pathway and Network Analysis

IPA was used to explore biological pathways associated with differential gene expression between participants with and without any neck disability. The top canonical pathways include: Interferon alpha/beta signaling (B-H *p*-value = 2.31 × 10^−11^; 17/76 genes, 22.4% overlap), Iron homeostasis signaling pathway (B-H *p*-value = 5.65 × 10^−7^; 17/145 genes, 11.7% overlap), Erythrocytes take up oxygen and release carbon dioxide (B-H *p*-value = 1.16 × 10^−5^; 5/7 genes, 71.4% overlap), Erythrocytes take up carbon dioxide and release oxygen (B-H *p*-value =1.80 × 10^−4^; 5/11 genes, 45.5% overlap), and Transcriptional Regulation of Granulopoiesis (B-H *p*-value = 5.83 × 10^−4^; 8/51 genes, 15.7% overlap).

**Figure 3:**
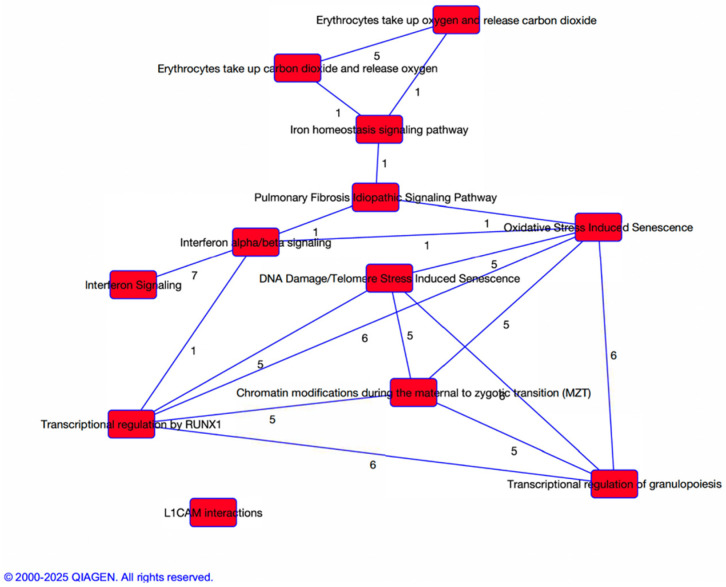
IPA identified top canonical pathway enriched among genes differentially expressed in relation to neck disability. The numbers adjacent to each pathway indicate the number of genes shared between the pathways, reflecting molecular overlap and potential crosstalk among biological processes. This figure highlights key mechanisms such as cellular stress responses, immune signaling, erythrocyte function, and transcriptional regulation, suggesting interconnected molecular pathways underlying neck disability.

Evaluation of the interaction between the pathways revealed functional overlap among pathways related to interferon signaling, iron homeostasis, and blood cell development suggesting strong molecular connectivity in functions such as hematological and immunological disease, cellular movement, and connective tissue disorders (Figure 3).

## 4. Discussion

This study provides novel insights into the molecular and clinical landscape of neck disability in OPSCC survivors, with a particular focus on neck pain measured via NDI. Despite comparable sociodemographic and clinical characteristics across clinical cohorts, patients reporting any level of neck disability exhibited notable gene expression profiles compared to those who reported no disability. Differential gene expression analysis revealed 24 significantly dysregulated genes with clustering patterns that aligned with self-reported neck disability status. Pathway enrichment and network analyses further implicated interferon signaling, iron homeostasis, and erythrocyte gas exchange patterns, highlighting potential biological mechanisms underlying post-radiation tissue remodeling and neuromuscular dysfunction. Findings of this exploratory study suggest that self-reported neck pain and disability may reflect a broader biological phenotype beyond localized fibrotic changes captured by traditional clinical grading. Clinically, these insights may inform the development of biomarker-driven strategies for early identification, risk stratification, and targeted interventions to mitigate symptom burden associated with neck disability in OPSCC survivors.

The composition of the cohorts was comparable in age, race, and tobacco/alcohol use, significant differences emerged in marital status, neck disability, and self-reported pain. Although the differences in neck disability and self-reported pain were expected, as these are signs and symptoms of worsening degree of radiation-induced fibrosis, the differences in marital status were interesting as they were not directly related to the cancer or cancer treatment. In our study, we found that as fibrosis grade increased, so did the number of people who were not married. It has previously been shown that not only are married patients less likely to report neck disability, but might also experience a survival benefit as they have been shown to have less metastatic disease and are more likely to receive adequate treatment [13,34,35]. These findings suggest that social support and physical function may be important correlates of post-treatment disability in OPSCC survivors and warrants further research. The significant association between NDI and pain highlights the ability of the NDI to capture perceived pain in our cohort of patients. It is important to note that the differences observed occurred in the absence of significant variation in tumor stage or HPV status, reinforcing the hypothesis that neck disability may be driven by biological and psychosocial factors beyond initial disease severity.

Differential gene expression analysis revealed 24 significantly dysregulated genes associated with neck pain and disability in OPSCC survivors, spanning key biological domains including immune regulation, hematopoiesis, neuronal function, and transcriptional control. Notably, genes such as *IFI27*, *GPR15*, and *ILDR2* were upregulated, implicating heightened interferon signaling and immune activation in patients with neck disability [36,37,38]. Concurrently, downregulation of neuronal genes like *NEFL*, *FEZ1*, and *PRICKLE2* suggest potential disruptions in neuromuscular integrity and sensory processing [39,40,41,42]. The upregulation of *HBG2* and *MAP3K6*, both involved in hematopoiesis and iron metabolism, may reflect compensatory vascular or oxygenation responses to tissue stress [43,44]. These transcriptomic patterns align with pathway enrichment results, reinforcing the hypothesis that neck disability reflects a multifaceted biological phenotype involving immune, vascular, and neuromuscular dysfunction rather than isolated fibrotic changes.

In addition to the previously highlighted immune and neuronal genes, the heatmap analysis revealed differential expression of transcriptional regulators and cellular function genes that may contribute to the biological complexity of neck pain and disability. Downregulation of *ZNF732* and *POLR2K* suggest altered transcriptional control mechanisms, potentially affecting cellular stress responses and repair pathways [45,46]. Genes such as *AKR1C3* and *PID1*, involved in tissue distributions and metabolic regulation, were also dysregulated, pointing to possible disruptions in local energy metabolism and tissue remodeling [47,48,49]. The upregulation of *EPB41L3*, a gene linked to cytoskeletal structure and neuronal stability, may reflect compensatory responses to neuromuscular strain [50]. Together, these findings expand the biological scope of neck pain and disability beyond immune and pain signaling, implicating transcriptional, metabolic, and structural pathways in the chronic sequelae experienced by HNC survivors.

Pathway enrichment analysis further contextualized the transcriptomic findings, revealing significant involvement of interferon alpha/beta signaling, iron homeostasis, and erythrocyte gas exchange, pathways that may underlie the persistent symptoms reported by OPSCC survivors with neck disability. The identification of oxidative stress-induced senescence and DNA damage/telomere stress pathways suggests that chronic cellular stress and impaired regenerative capacity may contribute to neuromuscular dysfunction [51]. Additionally, transcriptional regulation pathways such as *RUNX1*-mediated granulopoiesis and chromatin modification during the maternal-to-zygotic transition point to broader epigenetic and hematopoietic shifts [52,53]. These findings support a model in which neck pain and disability reflect not only localized tissue damage but also systemic biological responses involving immune activation, vascular remodeling, and cellular aging processes.

Notably, several genes identified in the transcriptomic analysis appear to be involved in multiple enriched pathways, suggesting they may serve as key regulatory nodes in the biological response to radiation and neck disability. For example, *IFI27* and *GPR15* are implicated in both interferon signaling and immune-related senescence pathways, highlighting their potential role in sustaining chronic inflammation and immune dysregulation [38,54]. Similarly, genes like *HBG2* and *MAP3K6*, which contribute to iron homeostasis and erythrocyte gas exchange, also intersect with pathways related to oxidative stress and hematopoiesis [55,56]. The recurrence of these genes across functionally related biological processes suggest that neck disability may arise from a convergence of immune, vascular, and metabolic dysfunctions rather than isolated pathway activation. This integrative view supports the development of multitargeted therapeutic strategies and reinforces the value of transcriptomic profiling in uncovering complex, overlapping mechanisms of survivorship burden.

It has previously been shown that although most changes in pain perception are likely due to alterations in nerve impulse transmission pathways, whole blood transcriptomic profiles can differentiate vulnerability to chronic low back pain [57]. In this study, the authors found significant enrichment for known pain genes, supporting the feasibility of detecting pain-related molecular signatures in blood, even though the mechanisms may be indirect or reflect systemic correlates of pain processing. In our study, the lack of enrichment for pain genes may reflect differences in pain type. Our cohort experienced radiation-induced musculoskeletal pain, which often involves fibrosis, vascular remodeling, and localized inflammation, whereas chronic low back pain may stem from mechanical dysfunction or systemic inflammatory conditions, and its molecular signature in blood may reflect different pathways. Another difference might be that our analysis was guided by subjective self-reported neck disability, which reflects the impact of pain on daily functioning, whereas the Dorsey et al. study incorporated both subjective and objective assessments. Importantly, our study did not exclude participants taking opioids, whereas Dorsey et al. did. This difference may influence the peripheral transcriptomic profile, as opioid use can modulate immune and inflammatory gene expression.

Findings from this study provide evidence that neck pain and disability in OPSCC survivors are possibly linked to molecular profiles marked by immune activation, neuronal disruption, and vascular-metabolic dysregulation. Transcriptomic profiling combined with pathway enrichment analysis revealed not only individual gene dysregulation but also recurring molecular signatures across key biological pathways—including interferon signaling, iron homeostasis, and erythrocyte gas exchange—suggesting a coordinated biological response to radiation-induced tissue stress that may underlie chronic pain and functional impairment. These molecular distinctions underscore the importance of integrative approaches that pair patient-reported outcomes (PROs) with transcriptomic data to better characterize post-treatment sequelae and uncover hidden dimensions of survivorship burden. Future research should explore whether these gene expression patterns can serve as predictive biomarkers and whether they are modifiable through targeted therapies or rehabilitation strategies. Moreover, deeper understanding of the interplay between interferon signaling, iron metabolism, and erythrocyte function may open new therapeutic avenues to mitigate neuromuscular complications and enhance long-term survivorship care.

### 4.1. Implications for Clinical Practice

This study identified candidate genes and pathways that may serve as therapeutic targets and biomarkers for symptom burden and rehabilitation response in OPSCC. Genes linked to neuroinflammation, and neuromuscular function could inform risk stratification and early intervention for patients at higher risk of persistent neck pain and disability. Integrating transcriptomic data with PROs supports a personalized approach to survivorship care, enabling tailored physical therapy, pharmacologic treatment, and supportive services. These findings align with precision health goals and emphasize the role of symptom biology in improving QOL for OPSCC survivors. If replicated, these results could inform precision care strategies by combining clinical, demographic, behavioral, and gene expression data to characterize individual symptom experiences and guide self-management. Patients with gene dysregulation linked to severe pain risk may benefit from targeted education and early intervention options to prevent or reduce symptom burden.

### 4.2. Limitations and Future Directions

In this exploratory analysis, the small sample size limited our ability to break up the categories of neck disability and pain into mild, moderate, and severe, instead we used the bivariate categories of any pain or any neck disability versus no pain or no neck disability, limiting our ability to determine differential gene expression related to pain severity. Also, as this was a cross-sectional study, we did not have the ability to look at intra-patient pain duration. We do recognize that there is overlap between NDI grouping and treatment timing. However, the primary objective of this exploratory study was to explore transcriptomic correlates of symptom burden, not to model treatment effects per se. While treatment timing is inherently linked to symptom development, our analysis focused on identifying molecular features associated with dysfunction regardless of treatment stage. Disentangling treatment effects from symptom-related molecular changes is challenging in cross-sectional designs. In future studies, we plan to pursue longitudinal sampling to better isolate temporal effects. Moreover, we acknowledge that the dichotomization of continuous variables such as NDI and pain score could lead to information loss and reduced statistical power. However, we chose this this approach based on the exploratory nature of the study and considering the modest sample size. The threshold that we used for dichotomization are grounded in established clinical cutoffs that reflect meaningful symptom burden. We also recognize that it is possible that our results may also be influenced by residual RNA from erythroid or reticulocyte populations; however, in the context of gene expression, we do not see a uniform expression level of these genes (e.g., *HBG2*) but rather an enrichment in specific subgroups, suggesting a potential biological relevance rather than a universal artifact. Additionally, our sample consisted mainly of White males over the age of 60, limiting the ability to generalize the results. Future studies should validate these findings in larger, longitudinal cohorts and explore functional roles of identified genes through in vitro or in vivo models. Multi-omics approaches, including epigenomics and proteomics, may further elucidate the complex biology of neck pain and disability in OPSCC. Ultimately, translating these insights into clinical tools could improve symptom prediction, monitoring, and intervention.

## Figures and Tables

**Table 1:** Participant characteristics.

Characteristic	No Fibrosis (n = 13)	Mild Fibrosis (n = 20)	Moderate/ Severe Fibrosis (n = 18)	*p*-Value
**Age (years, mean ± SD)**	63.69 ± 7.04	68.80 ± 7.36	66.83 ± 10.98	0.271
**Race: White (count, %)**	12 (92.3%)	17 (85.0%)	17 (94.4%)	0.593
**Sex: Male (count, %)**	12 (92.3)	18 (90.0)	12 (66.7)	0.094
**Female (count, %)**	1 (7.7)	2 (10.0)	6 (33.3)	
**Marital Status: Married (count, %)**	11 (84.6)	15 (75.0)	8 (44.4)	0.034 *
**Other ^a^ (count, %)**	2 (15.4)	5 (25.0)	10 (55.6)	
**Tobacco Use (ever): Yes (count, %)**	7 (53.8)	10 (50.0)	8 (44.4)	0.870
**No (count, %)**	6 (46.2)	10 (50.0)	10 (55.6)	
**Alcohol Use (ever): Yes (count, %)**	11 (84.6)	18 (90.0)	12 (66.7)	0.176
**No (count, %)**	2 (15.4)	2 (10.0)	6 (33.3)	
**Tumor Stage: T1/T2 (count, %)**	8 (61.5)	18 (90.0)	12 (66.7)	0.119
**T3/T4 (count, %)**	5 (38.5)	2 (10.0)	6 (33.3)	
**HPV Status: Yes (count, %)**	12 (92.3)	16 (80.0)	12 (66.7)	0.225
**No (count, %)**	1 (7.6)	4 (20.0)	6 (33.3)	
**Neck Disability: None (count, %)**	12 (92.3)	19 (95.0)	8 (44.4)	0.003 *
**Any (count, %)**	1 (7.7)	1 (5.0)	10 (55.6)	
**Self-reported Pain: None (count, %)**	12 (92.3)	13 (65.0%)	4 (22.2)	<0.001 *
**Any (count, %)**	1 (7.7)	7 (35%)	14 (77.8)	
**Area Deprivation (mean ± SD)**	59.38 ± 29.37	59.05 ± 23.12	64.22 ± 18.59	0.884

* Statistically significant difference; ^a^ divorced/living with partner/widowed/single.

**Table 2:** Significant genes.

Gene Name	Gene Symbol	Adjusted *p*-Value	log_2_ Fold Change	Gene Function
**Killer Cell Lectin Like Receptor F1**	*KLRF1*	0.002	−1.30	Immune Function
**Core−Binding Factor Subunit Beta**	*CBFB*	0.002	−0.35	Hematopoiesis
**Zinc Finger Protein 600**	*ZNF600*	0.003	−1.09	Transcriptional Regulation
**Mitogen−Activated Protein 3 Kinase Kinase Kinase 6**	*MAP3K6*	0.017	0.71	Hematopoiesis
**G Protein−Coupled Receptor 15**	*GPR15*	0.017	1.94	Immune Function
**SAM And SH3 Domain Containing 1**	*SASH1*	0.017	1.16	Immune Function
**Neurofilament Light Chain**	*NEFL*	0.017	−1.48	Neuronal Function
**Hemoglobin Subunit Gamma 2**	*HBG2*	0.017	4.21	Hematopoiesis
**Fasciculation And Elongation Protein Zeta 1**	*FEZ1*	0.017	−1.48	Neuronal Function
**Interferon Alpha Inducible Protein 27**	*IFI27*	0.018	3.01	Immune Function
**Erythrocyte Membrane Protein Band 4.1 Like 3**	*EPB41L3*	0.022	0.75	Neuronal Function
**Prickle Planar Cell Polarity Protein 2**	*PRICKLE2*	0.022	−2.08	Neuronal Function
**Centrosomal Protein 126**	*CEP126*	0.028	−0.98	Cellular Function
**Zinc Finger Protein 732**	*ZNF732*	0.030	−1.52	Transcriptional Regulation
**Zinc Finger CCHC−Type Containing 17**	*ZCCHC17*	0.035	−0.41	Transcriptional Regulation
**Immunoglobulin Like Domain Containing Receptor 2**	*ILDR2*	0.036	2.55	Immune Function
**GSK3B Interacting Protein**	*GSKIP*	0.036	−0.94	Neuronal Function (neurite outgrowth)
**Immunoglobulin Superfamily Member 10**	*IGSF10*	0.041	2.03	Neuronal Function
**CXXC Finger Protein 4**	*CXXC4*	0.042	−1.81	Hematopoiesis (Fe) Wnt signaling
**Zinc Finger Protein 595**	*ZNF595*	0.043	−1.37	Transcriptional Regulation
**Protocadherin Gamma Subfamily B, 7**	*PCDHGB7*	0.043	−1.42	Neuronal Function
**Aldo−Keto Reductase Family 1 Member C3**	*AKR1C3*	0.047	−1.11	Cellular Function (tissue distribution)
**HEN Methyltransferase 1**	*HENMT1*	0.047	−0.53	Transcriptional Regulation
**RNA polymerase II, I and III Subunit K**	*POLR2K*	0.047	−1.12	Transcriptional Regulation

## Data Availability

De-identified data can be made available upon request for research purposes to qualified individuals within the scientific community.
